# Erster deutscher Expertenkonsens zu Telemedizin in der Urologie

**DOI:** 10.1007/s00120-026-02800-z

**Published:** 2026-03-06

**Authors:** Severin Rodler, Julian Risch, Marie-Luise Weiss, Sami Leyh-Bannurah, Moritz von Bueren, Fabian Siegel, Andreas Meißner, Alexander Piotrowski, Maximilian Bauser, Dirk M. Potempa, Mattis Franke, Andreas W. Schneider, Edwin Hermann, Johannes von Bueren, Jakob Kohler, Philipp Nuhn, Christian Wülfing, Ulrich Witzsch, Julian Struck, Hendrik Borgmann

**Affiliations:** 1 Klinik für Urologie, Universitätsklinikum Schleswig-Holstein, Arnold-Heller-Straße 3, 24103 Kiel, Deutschland; 2 Arbeitskreis Künstliche Intelligenz und Digitalisierung der Deutschen Gesellschaft für Urologie, Düsseldorf, Deutschland; 3 Arbeitskreis Informationstechnologie und Dokumentation (IT@Doc), Düsseldorf, Deutschland; 4 Martini-Klinik Prostate Cancer Center, University Hospital Hamburg-Eppendorf, Hamburg, Deutschland; 5 Urologische Facharztpraxis, Freiburg, Deutschland; 6 Department of biomedical informatic, Universitätsklinikum Mannheim, Mannheim, Deutschland; 7 Department of Urology, Center for Reproductive Medicine, Amsterdam University Medical Center (UMC), Amsterdam Reproduction and Development Research Institute, University of Amsterdam, Amsterdam, Niederlande; 8 Klinikum Bad Hersfeld GmbH, Bad Hersfeld, Deutschland; 9 Medizinische Klinik und Poliklinik 1, Uniklinikum Würzburg, Würzburg, Deutschland; 10 Uro Zentrum GAP PartG, Garmisch-Partenkirchen, Deutschland; 11 Klinik für Urology, Asklepios Klinik Altona, Hamburg, Deutschland; 12 Praxis für Urologie in Winsen/Luhe, Buchholz, Deutschland; 13 Privatpraxis Herrmann & Secker, Münster, Deutschland; 14 Accessus Science Technologies GmbH, München, Deutschland; 15 Urologische Privatpraxis Ulrich Witzsch, Sulzbach, Deutschland; 16 Klinik für Urologie, Universitätsklinikum Brandenburg an der Havel, Brandenburg an der Havel, Deutschland

**Keywords:** Telemedizin, Ausbildung, Delphi Konsensus, Synchron, Asynchron, Telemedicine, Training, Delphi Consensus, Synchronous, Asynchronous

## Abstract

**Hintergrund:**

Telemedizin findet breite Anwendung in der Urologie, wird allerdings in Leitlinien bisher nicht ausreichend berücksichtigt.

**Methodik:**

Es wurde ein zweistufiger Expertenkonsensus durchgeführt, wobei in zwei Runden Statements von Experten aus der Urologie evaluiert wurden. Zwischen den Runden erfolgten entweder virtuelle oder vor Ort Besprechungen. Statements mit 75 % Zustimmung in der finalen Runde wurden als angenommen gewertet.

**Ergebnisse:**

15 Experten nahmen an der ersten Abstimmungsrunde teil, wobei Konsensus in 20/21 Statements erzielt werden konnte. Nach erneuter Diskussion haben 11 Experten 22/23 Statements angenommen.

**Schlussfolgerung:**

Telemedizin eröffnet neue Versorgungsmodelle in der Urologie; entscheidend für die Umsetzung sind Anpassungen bei Vergütung und Ausbildung aller Gesundheitsberufe.

**Graphic abstract:**

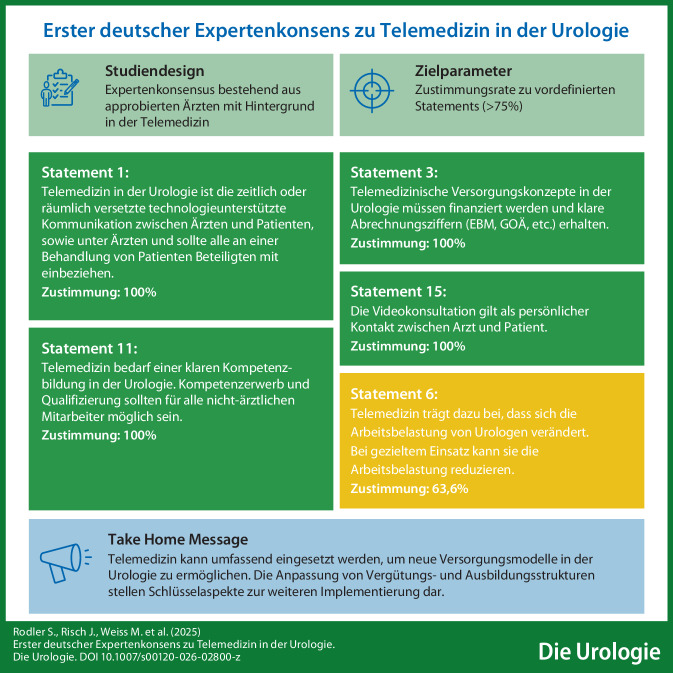

**Zusatzmaterial online:**

Die Online-Version dieses Artikels (10.1007/s00120-026-02800-z) enthält eine weitere Tabelle.

## Abstract (en)

**Background:**

Telemedicine is widely used in urology, but it has so far been insufficiently considered in clinical guidelines.

**Methods:**

A two-stage expert consensus was conducted in which urology experts evaluated statements in two rounds. Between rounds, discussions took place either virtually or in person. Statements with 75% agreement in the final round were considered to be accepted.

**Results:**

Fifteen experts participated in the first round of voting, achieving consensus on 20 out of 21 statements. After renewed discussion, 11 experts accepted 22 out of 23 statements.

**Conclusion:**

Telemedicine opens up new models of care in urology. Successful implementation requires adjustments in reimbursement and the training of all healthcare professionals.

Telemedizin findet breite Anwendung in der Urologie, aber wird bisher nicht in Leitlinien berücksichtigt. Da bisher prospektive Versorgungsstudien fehlen, soll anhand eines Expertenkonsensus erste Evidenz und Ausganspunkt für Studien geschaffen werden.

## Hintergrund

Telemedizin ist die federführende Technologie, durch deren Einsatz in der Urologie die Arzt-Patienten-Beziehung signifikant weiterentwickelt wird [1]. Unter Telemedizin versteht man den Austausch von medizinischen Informationen über eine elektronische Kommunikation zwischen verschiedenen Standorten mit dem Ziel, die Gesundheit eines Patienten zu verbessern [2]. Aktuell versteht man zunehmend auch die Kommunikation zwischen Patienten und „professionellen Gesundheitsanbietern“ im Generellen unter diesem Begriff.

Grundsätzlich werden zwei Arten von Telemedizin unterschieden, die in jeweils sehr unterschiedlichen Behandlungskontexten eingesetzt werden können. Synchrone Telemedizin beschreibt alle direkten Arzt-Patienten-Interaktionen. Sie wird beispielsweise über Telefon oder Videotelefon durchgeführt. Asynchrone Telemedizin dagegen beschreibt die zeitlich versetzte Interaktion von Patienten mit Anbietern von Gesundheitsleistungen [3].

Erst die Kombination und Integration beider Arten von Telemedizin in klinische Abläufe führt zu einer breiten Nutzung innerhalb der Ärzte- sowie Patientenschaft. Während der COVID-19-Pandemie („coronavirus disease 2019“) konnte gezeigt werden, dass der breite Einsatz von Telemedizin zur Überbrückung von Distanzen zwischen Ärzten und Patienten bei gleichzeitiger Wahrung der Hygieneauflagen möglich ist [1]. Neuere Forschungsarbeiten untersuchen dahingehend zunehmend, wie Telemedizin die Versorgung von Patienten nachhaltig verändert. Dabei stellen neben der reinen Umsetzbarkeit von Telemedizin inklusive Sicherheit der Patienten und Nebenwirkungen auch Zeit- und Kostenersparnisse sowie ökologische Aspekte weitere Endpunkte in Studien dar [4].

Telemedizin wird bereits in der Urologie aber auch in vielen anderen medizinischen Fachgebieten genutzt. Sie kann dabei den Alltag der Mediziner sowie Patienten gleichermaßen unterstützen und entlasten, und doch findet sie bisher keine Berücksichtigung in deutschen urologischen Leitlinien [5]. Dies liegt nicht an einem fehlenden Nachweis von Nutzen und Wirksamkeit der Telemedizin, sondern ist vielmehr der Komplexität großangelegter Versorgungsstudien sowie den komplexen, häufig sehr zeitintensiven Prozessen der Leitlinienüberarbeitung geschuldet. Die Ergebnisse des vorliegenden Expertenkonsensus sollen dabei helfen, diese Lücke zu schließen und den aktuellen Stellenwert der Telemedizin im Bereich der Urologie wiedergeben. Ein Fokus lieg dabei auf der Definition von Telemedizin, der klinischen Anwendung und sinnvollen Nutzung sowie der Identifizierung von Faktoren, die eine weitere Implementierung von Telemedizin in den urologischen Alltag unterstützen können.

## Methoden

In einem ersten Schritt wurde eine Literaturrecherche zum Thema Telemedizin in der Urologie durchgeführt. Hierzu wurden die Mesh-Terms „telemedicine“, „telehealth“ und „urology“ verwendet. Die Suchergebnisse wurden für die Hintergrundrecherche zur Vorbereitung des ersten Online-Treffens verwendet.

Experten wurden über den Arbeitskreis „Informationstechnologie und Dokumentation (AK IT@DOC)“ der DGU sowie aus im Bereich Telemedizin tätigen Urologen rekrutiert. Als Experten wurden Personen gewertet, die sich mit Telemedizinanwendungen in der Urologie entweder in der praktischen Umsetzung oder theoretisch in der Forschung auseinandersetzen. Alle Experten mussten approbierte Ärzte sein.

Zunächst fand ein Online-Treffen der Teilnehmer statt (Zoom Communications, San José, Kalifornien, USA). Im Rahmen des Onlinemeetings wurden basierend auf den Ergebnissen der Literaturrecherche Arbeitspakete definiert. Die Arbeitspakete wurden im Nachgang zu dem Meeting in Statements umgewandelt. Im Anschluss wurden innerhalb eines Zeitraums von 2 Wochen ein digitales Rating vorgenommen (SurveyMonkey Corp., San Mateo, Kalifornien, USA). Antwortmöglichkeiten beinhalteten: 1) ich stimme überhaupt nicht zu, 2) ich stimme nicht zu, 3) ich stimme weder zu noch stimme ich nicht zu, 4) ich stimme zu, 5) ich stimme voll und ganz zu. Antwortoption 4 und 5 wurde als Zustimmung gewertet. Es war möglich, auch Kommentare zur Änderung des Statements abzugeben. A priori wurde festgesetzt, dass mindestens 75 % aller Experten einem Statement zustimmen müssen, um Konsensus zu erzielen.

**Abb. 1:**
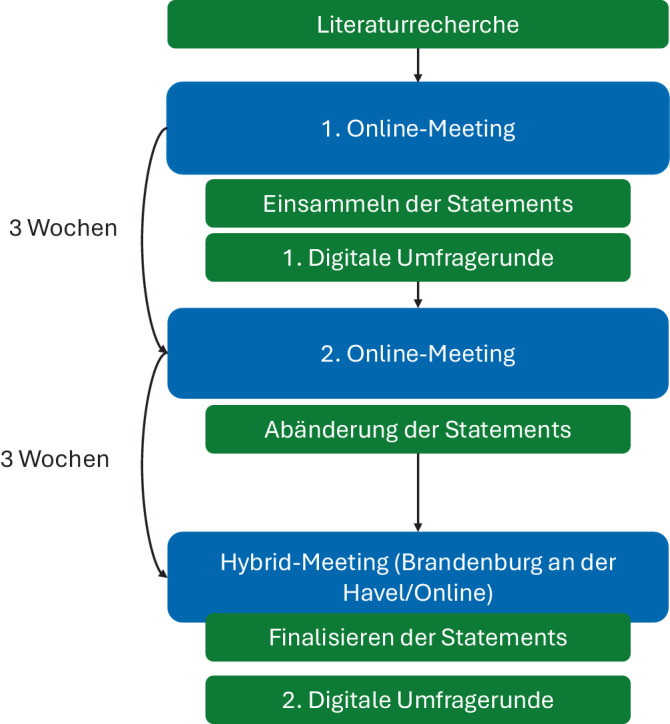
Ablauf der Konsensusfindung

Basierend auf den Ergebnissen der ersten Runde fand eine erneute digitale Besprechung der Ergebnisse und teilweise Umformulierung bzw. Klarstellung von Statements statt. Auf dem 2. Digital Health Summit in Brandenburg an der Havel, Deutschland, fand dann die finale Besprechung der Statements und Festlegung des finalen Wortlautes statt. Teilnehmer, die nicht persönlich teilnehmen konnten, nahmen digital teil (Zoom Communications, San José, Kalifornien, USA). Im Rahmen der zweiten digitalen Umfrage (SurveyMonkey Corp., San Mateo, Kalifornien, USA) wurde im Anschluss das Ergebnis der finalen Abstimmungsrunde erzielt (Ablauf der Konsensusfindung s. Abb. 1).

Ergebnisse werden als Prozentzahlen der Zustimmungsquote dargestellt. Darüber hinaus wurden keine statistischen Verfahren angewendet.

## Ergebnisse

Sechzehn präselektionierte Experten nahmen am ersten Online-Meeting teil und definierten basierend auf der Literaturrecherche die Statements der ersten Konsensusrunde (Expertenzusammensetzung s. Tab. 1, Statements s. Zusatztabelle 1). Die anschließende digitale 1. Umfragerunde wurde von 15 Experten beantwortet und erbrachte Konsensus in 20/21 Statements, wobei Kommentare zu jedem Statement zur weiteren Schärfung oder bei Unklarheit in der Formulierung abgegeben wurden.

**Tab. 1:** Charakteristika der Expertengruppe in der zweiten Konsensusrunde

	*n* = 11
*Alter (Jahre)*	
Median	45
Range	30–63
*Qualifikation*	
Approbation	11
Promotion	11
Facharzt für Urologie	9
Habilitation	4
*Tätigkeitsgebiet*	
Universitätsklinikum	4
Praxis/MVZ	3
Klinik	3
Telemedizinunternehmen	1

An der 2. Runde des modifizierten Delphi-Verfahrens nahmen 11 Experten teil (Statements s. Tab. 2). 22/23 Statements erreichten dabei die prädefinierte Zustimmungsrate. Statement 6 erreichte dabei nicht die notwendige Konsensusrate in der finalen Abstimmung.

**Tab. 2:** Konsensusstatements in der zweiten Abstimmungsrunde

Nummer	Frage	Zustimmung (%)
1	Telemedizin in der Urologie ist die zeitlich oder räumlich versetzte technologieunterstützte Kommunikation zwischen Ärzten und Patienten, sowie unter Ärzten und sollte alle an einer Behandlung von Patienten Beteiligten mit einbeziehen	100
2	Vor dem Hintergrund einer drohenden Mangelversorgung in der Urologie ist es zwingend notwendig, synchrone und asynchrone telemedizinische Konzepte anzuwenden, die bisherige urologische Behandlungsprinzipien ergänzen und weiterentwickeln	90,9
3	Telemedizinische Versorgungskonzepte in der Urologie müssen finanziert werden und klare Abrechnungsziffern (EBM, GOÄ etc.) erhalten	100
4	Die telemedizinische Versorgung sollte der Präsenspflichtzeiten von Kassenärzten angerechnet werden. Hierbei sind Patienten aus dem jeweiligen Gebiet des Kassensitzes zu behandeln	81,8
5	In der Urologie ist aufgrund der demographischen Entwicklung und der damit steigenden Inzidenzen urologischer Diagnosen mit einer starken Steigerung der Patientenzahlen zu rechnen. Die telemedizinische synchrone wie asynchrone Behandlung muss daher als Therapie- und Behandlungsform in der Urologie aktiv eingesetzt werden als Alternative zur Präsensvorstellung	100
6	Telemedizin trägt dazu bei, dass sich die Arbeitsbelastung von Urologen verändert. Bei gezieltem Einsatz kann sie die Arbeitsbelastung reduzieren	63,6
7	Bei der Anwendung von Telemedizin in der Urologie ist der Arbeitsschutz des Arztes oder Mitarbeitern genauso wie in der konventionellen Versorgung zu gewährleisten. Permanenter Zugang von Patienten zu medizinischem Personal muss eingegrenzt werden	90,9
8	Telemedizin ist Bestandteil des medizinischen Fortschritts und sorgt auch im Berufsalltag der Urologen für mehr Flexibilität und einen modernen Arbeitsplatz	90,9
9	Telemedizin (synchron und asynchron) kann Teile von Behandlungspfaden oder komplette Behandlungen in der Urologie effizienter und schneller gestalten	90,9
10	Telemedizin bedarf einer klaren Kompetenzbildung in der Urologie. Die Ausbildung sollte daher sowohl im Studium als auch in der Facharztausbildung sowohl theoretisch als auch praktisch erfolgen	90,9
11	Telemedizin bedarf einer klaren Kompetenzbildung in der Urologie. Kompetenzerwerb und Qualifizierung sollten für alle nicht-ärztlichen Mitarbeiter möglich sein	100
12	Telemedizin und deren Durchführung basiert bis zur Aufnahme in Leitlinien auf Expertenkonsens, der wie eine Leitlinienempfehlung akzeptiert wird	90,9
13	Tools zum Einsatz von Telemedizin sind vorrangig Videokonsultationen sowie medizinische Fragebögen (sofern anwendbar validiert). Beim Einsatz von unstrukturierten Tools (wie z. B. Anamnesebögen) sollte zusätzlich eine genaue ärztliche Prüfung durchgeführt werden	90,9
14	Die Videokonsultation entspricht dem fachlichen Standard in der Urologie	81,9
15	Die Videokonsultation gilt als persönlicher Kontakt zwischen Arzt und Patient	100
16	Die Behandlung eines urologischen Patienten kann telemedizinisch erfolgen, sofern diese unter Wahrung der ärztlichen Sorgfalt erfolgt und der Art und Weise der Befunderhebung, der Besonderheit der Beratung, Behandlung und Dokumentation durchgeführt wird	90,9
17	Medizinische Fragebögen gehören zum Standard telemedizinischer asynchroner Anamnese und Behandlung: Ein anamnestischer asynchroner Fragebogen:– muss ärztlich ausgewertet werden– stellt einen Patienten-Arzt-Kontakt dar	90,9
18	Auf Basis der Patientenhistorie und urologischen Indikation liegt es im Ermessen des Arztes, ob die Anamnese synchron oder asynchron erfolgt	90,9
19	Im Rahmen der telemedizinischen Versorgung können neue Daten gewonnen werden (sowohl synchrone als auch asynchrone Telemedizin). Diese Daten sollten strukturiert der Versorgungsforschung, unter Wahrung der Persönlichkeitsrechte des Patienten, zugänglich sein	90,9
20	Fachgesellschaften (z. B. DGU, BDU gemeinsam) sollen aktiv Standards für die telemedizinische Infrastruktur definieren	81,8
21	Fachgesellschaften unter Führung der DGU sollen aktiv an urologiespezifischen Telemedizinguidelines arbeiten	100
22	Bei sinnvollem Einsatz von Telemedizin erscheint eine Limitierung der telemedizinischen Vorstellungen kontraproduktiv in der Etablierung	81,8
23	Telemedizin hilft in der Urologie, Ressourcen zu sparen	81,8

**Abb. 2:**
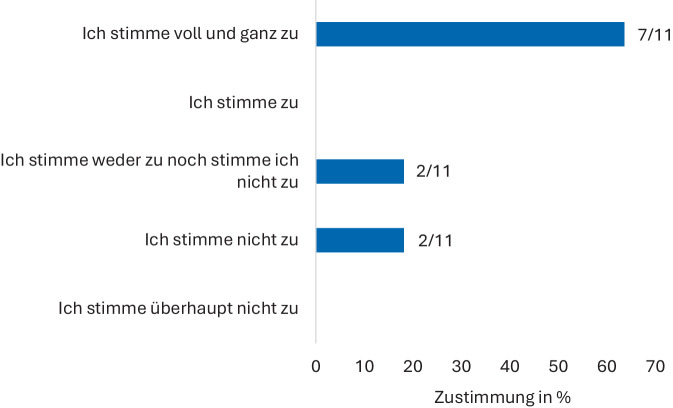
Ergebnisverteilung des abgelehnten Statements

Die Statements 1, 3, 5, 11, 15 und 21 erhielten die volle Zustimmung aller Experten (11/11). Die Statements 2, 4, 7, 8, 10, 12–14, 16, 18–20, 22, 23 erzielte keine Gegenstimmen, jedoch Expertenmeinungen, die weder zu– noch dagegen stimmten. Die Statements 6, 9 und 17 wurden jeweils von mindestens einem Experten abgelehnt. Statement 6 erzielte dabei keinen Konsensus (Zustimmung: 63,6 %). Diesem Statement stimmten 4/11 (36,4 %) Experten nicht zu, wobei 2/11 (18,2 %) Experten unentschieden waren (Tab. 2 und Abb. 2).

## Diskussion

Telemedizin ist eine weitverbreitete, technologiegestützte Kommunikationsmöglichkeit in der Urologie. Der aktuelle Konsensus bietet eine umfassende Definition von Telemedizin sowie Anwendungsempfehlungen für die Urologie. Ferner verdeutlicht er die Notwendigkeit zur Optimierung der Kostenerstattung und Integration in die urologische Aus- und Weiterbildung.

Das Schließen von Versorgungslücken und Finden von Antworten auf den demographischen Wandel stellen das Gesundheitssystem und insbesondere die Urologie vor große Herausforderungen. Es wird erwartet, dass die Anzahl der urologischen Patienten massiv ansteigt. Gleichzeitig plant ein relevanter Anteil an Urologen, in den nächsten Jahren in den wohlverdienten Ruhestand zu gehen, wobei von bis zu 20 % der aktuell klinisch tätigen Urologen über 5 Jahre ausgegangen wird [6]. Diese Lücke muss effektiv geschlossen werden, um weiterhin eine qualitativ hochwertige, urologische Versorgung insbesondere auch in ländlichen Gebieten sicherstellen zu können. Für die Versorgung im ländlichen Bereich wurde Telemedizin bereits als eine Schlüssellösung identifiziert [7]. Die bisherigen Studien im Bereich Telemedizin beschränkten sich indes vorrangig auf die synchrone Telemedizin im Rahmen der COVID-19-Pandemie, um physische Distanzen zu überwinden [8]. Allerdings hat sich die asynchrone Telemedizin als ebenfalls effektiv erwiesen, um potenzielle Barrieren zwischen Behandlern und Patienten zu überwinden [9]. Das Expertengremium stimmt darin überein, dass die bestehende urologische Versorgung durch neue telemedizinische Konzepte ergänzt und erweitert werden sollte. Beispiele hierfür können virtuelle Versorgungsmodelle zum PSA-Screening oder Nachsorgemodelle bei onkologischen Erkrankungen sein. Der Einsatz telemedizinischer Werkzeuge sollte flexibilisiert werden, um so sowohl synchrone als auch asynchrone Lösungen anbieten zu können. Wichtig bleibt jedoch eine ärztliche Validierung, insbesondere in Anamnesefragen, um einen sicheren Einsatz zu ermöglichen. Die Auswertung solcher Tools durch den Arzt sollte aus Sicht der Experten als Arzt-Patienten-Kontakt gelten, mit potenzieller Auswirkung auf die Vergütung.

Die Finanzierung ist damit einer der Schlüsselaspekte in der Einführung und dauerhaften Etablierung telemedizinischer Anwendungen. Aktuell erfolgt ein Abschlag von 20–30 % auf die Grundpauschalen bei reiner telemedizinischer Behandlung, weshalb eine persönliche Vorstellung zumindest einmal im Quartal weiterhin ökonomischer insentiviert wird [10]. Zudem wurde in den letzten Jahren die telemedizinische Leistung künstlich verknappt. Bisher durften nur 30 % aller bekannten Patienten telemedizinisch behandelt werden [11]. Das Expertengremium stimmt überein, dass eine Beschränkung der telemedizinischen Leistung für eine Implementierung insgesamt hinderlich ist. Diese Grenze ist am 01.04.2025 gefallen. Nunmehr können 50 % aller Patienten dieser Versorgungsform zugeführt werden. Voraussetzung dabei ist, dass die Patienten mindestens einen persönlichen Arztkontakt in den drei Vorquartalen gehabt haben [11]. Neu eingeführt wurde auch ein Zuschlag von 3,72 € für bekannte Patienten, die in einem Quartal ausschließlich telemedizinisch behandelt werden [10].

Die Experten sind sich einig, dass sich bezüglich der Arbeitsbelastung von Urologen durch Integrierung von Telemedizin noch keine abschließende Aussage tätigen lässt. Die Kontroverse ist bereits von verschiedenen Autoren beschrieben worden, dass Telemedizin nicht zwangsläufig zu einer Verringerung der Arbeitsbelastung führen muss, sondern in einigen untersuchten Kollektiven zu einer Zunahmen beispielsweise der Arbeit außerhalb der eigentlichen Arbeitszeit führen kann [12]. Interessanterweise wurde während der COVID-19-Pandemie sogar eine Verschlechterung der Arbeitseffizienz festgestellt [13], wobei diese Studie nur synchrone Telemedizin einschließt. Weitere prospektive Studien sind daher insbesondere in diesem Forschungsgebiet notwendig, um den Effekt von Telemedizin auf die Arbeitsbelastung von Urologen zu verstehen.

Ausbildung und Weiterbildung wird als Schlüsselkatalysator für den zukünftigen Einsatz von Telemedizin gesehen. Aktuell fordern verschiedene Autoren ein strukturiertes Ausbildungscurriculum [14, 15]. Es ist jedoch noch unklar, welche Inhalte hier genau vermittelt werden sollen. Auch beschränkt sich die Forderung nach Ausbildung oft auf Urologen [16]. Die Mitglieder der Konsensusarbeitsgruppe stellen klar, dass dies nicht ausreicht, und alle an der medizinischen Versorgung beteiligten Berufsgruppen an der Integration der Telemedizin in die Ausbildung beteiligt werden sollten. Diese sollte beispielsweise bereits im Medizinstudium ansetzen und sowohl theoretische als auch praktische Inhalte umfassen. Es wird in den Statements gezielt vom Kompetenzerwerb gesprochen, um hervorzuheben, dass eben die spätere Übertragung auf neue Situation und damit Anwendung im Alltag möglich gemacht werden soll.

Eine Verknüpfung neuer technologischer Entwicklungen mit den bisherigen telemedizinischen Versorgungsmöglichkeiten wird aus Sicht der Experten die Integration beschleunigen und am Ende hoffentlich zu einer wirklichen Arbeitsentlastung führen. Am Beispiel von Large-language-Modellen konnte bereits gezeigt werden, dass sich diese neue Anwendungen an die Kommunikation und Sprachmöglichkeiten der Patienten anpassen können [17]. Dieser Einsatz von künstlicher Intelligenz wird von Patienten in der Urologie stark gewünscht [18] und hat damit das Potential, die Telemedizin als Werkzeug noch effizienter einzusetzen und damit die Arbeitsbelastung bei gleichzeitig optimierter Patientenversorgung zu reduzieren.

Schlussfolgernd stellt sich aus diesem Expertenkonsensus ein klarer Auftrag an die Gesundheitspolitik, Kostenträger sowie Verbände, die Rahmenbedingungen für eine dauerhafte Implementierung von Telemedizin in der Urologie zu schaffen. Als wichtige Ansatzpunkte werden dabei die Definition von technischen und medizinischen Standards für die Behandlung sowie eine wirtschaftliche Erstattung von Telemedizin aufgeführt. Innerhalb dieser Bandbreiten kann sich dann ein Ökosystem entwickeln, das sowohl Patienten in der Behandlung unterstützen als auch Gesundheitsanbieter in der Arbeitsbelastung entlasten kann.

Der vorliegende Expertenkonsens ist durch die begrenzte Anzahl an Experten und den rein explorativen Charakter in Ihrer Aussagekraft limitiert. Zudem nahmen nicht alle geladenen Experten an der zweiten Abstimmungsrunde teil. Zusätzlich kann der Auswahlprozess der Experten zu einem potenziellen Selektionsbias geführt haben. Da aber wie erwähnt der klinische Einsatz von Telemedizin bisher kein Bestandteil urologischer Leitlinien ist, stellen die in diesem Konsensus ausgesprochene Expertenmeinungen den Anfang für eine systematischen Evidenzanalyse und Aufarbeitung der Datenlage dar. Die sukzessive Aufarbeitung der Statements in hieran anknüpfenden Versorgungsstudien und die Übernahme in die entsprechenden krankheitsspezifischen Leitlinien wird künftig zur schrittweisen Akzeptanz und Implementierung von Telemedizin in die traditionelle Medizin und insbesondere Urologie führen.

## Fazit für die Praxis

- Der erste deutsche Expertenkonsens zu Telemedizin in der Urologie schließt eine Evidenzlücke in Ermangelung einer Integration in bestehende, deutsche urologische Leitlinien.
- Telemedizin kann umfassend eingesetzt werden, um neue Versorgungsmodelle in der Urologie zu ermöglichen.
- Die Anpassung von Vergütungs- und Ausbildungsstrukturen stellen Schlüsselaspekte zur weiteren Implementierung dar.

## Supplementary Information

Zusatztabelle 1: Fragen der ersten Konsensusrunde

## Data Availability

Alle Primärdaten sind vom Erstautor auf Nachfrage erhältlich.
