# Supplementary material for: First German expert consensus on telemedicine in urology
Source: Urologie. 2026 Mar 6;65(6):639–46. [Article in German] doi: 10.1007/s00120-026-02800-z (PMC13233988; doi:10.1007/s00120-026-02800-z)
Supplement: Supplementary file 1 — Zusatztabelle 1: Fragen der ersten Konsensusrunde [file 120_2026_2800_MOESM1_ESM.pdf]

Erster deutscher Expertenkonsens zu Telemedizin in der Urologie

S. Rodler et al.

Zusatztabelle 1: Fragen der ersten Konsensusrunde

Nummer	Frage	Zustimmung
1	Vor dem Hintergrund einer drohenden Mangelversorgung in der Urologie ist es zwingend notwendig, synchrone und asynchrone telemedizinische Konzepte zu entwickeln, die bisherige Behandlungsprinzipien ergänzen und weiterentwickeln.	93.3%
2	Telemedizinische Versorgungskonzepte in der Urologie müssen finanziert werden und klare Abrechnungsziffern (EBM) erhalten.	100%
3	Im Fachgebiet der Urologie ist aufgrund der demographischen Entwicklung der Gesellschaft und der steigenden Inzidenzen urologischer Diagnosen mit einer starken Steigerung der Patientenzahlen zu rechnen.Es erscheint daher sachgerecht den Fortschritt in der medizinischen Entwicklung mit der Telemedizin zu begegnen. Die synchrone wie asynchrone Behandlung muss daher als Therapie- und Behandlungsform medizinisch akzeptiert werden.	86.7%
4	Telemedizin kann die Arbeitsbelastung von Urologen reduzieren und Überlastung reduzieren.	60.0%
5	Telemedizin kann das Berufsleben von Urologen flexibilisieren und ermöglicht damit moderne Arbeitsplatzmodelle.	93.3%
6	Telemedizin (synchron und asynchron) kann Teile von Behandlungspfaden oder komplette Behandlungen in der Urologie effektiver und schneller machen.	93.3%
7	Telemedizin wird in der Urologie von Urologen,MFAs, Pflegekräften und allen direkt an der patientenbehandlung beteiligten Parteien abgebildet.	80%
8	Telemedizin bedarf einer klaren Kompetenzbildung in der Urologie. Die Ausbildung sollte daher sowohl im Studium als Kompetenz als auch in der Facharztausbildung thematisiert werden.	93.3%

9	Telemedizin bedarf einer klaren Kompetenzbildung in der Urologie. Kompetenzerwerb und Qualifizierung sollten für MFAs o. ä. Berufsgruppen möglich sein.	86.7%
10	Da die fachlichen Standards zur Erlangung ihrer rechtlichen Relevanz einer "allgemeinen Anerkennung" bedürfen, muss sich die inhaltliche Standard-Bestimmung zudem an dem Konsens- oder Mehrheitsprinzip ausrichten.	84.6%
11	Telemedizinischer Standard in der Urologie sind folgende Tools:- Videokonsultation für synchrone Telemedizin-Medizinisch validierter Fragebogen für asynchrone Telemedizin	100%
12	Die Videokonsultation entspricht dem fachlichen Standard in der Urologie.	78.6%
13	Die Videokonsultation gilt als persönlicher Kontakt zwischen Arzt und Patient.	93.3%
14	Die Aufklärung eines urologischen Patienten kann telemedizinisch erfolgen unter Wahrung der ärztlichen Sorgfalt insbesondere durch die Art und Weise der Befunderhebung, Beratung, sowie Dokumentation sowie nach Aufklärung des Patienten über die Besonderheiten der ausschließlichen Beratung über Kommunikationsmedien.	86.7%
15	Die Behandlung eines urologischen Patienten kann telemedizinisch erfolgen unter Wahrung der ärztlichen Sorgfalt insbesondere durch die Art und Weise der Befunderhebung, Beratung, Behandlung sowie Dokumentation sowie nach Aufklärung des Patienten über die Besonderheiten der ausschließlichen Beratung und Behandlung über Kommunikationsmedien.	93.3%
16	Ein anamnestischer asynchroner Fragebögen: - Muss medizinisch validiert sein- muss ärztlich ausgewertet werden-stellt einen Patienten-Arzt-Kontakt dar.	91.7%
17	Auf Basis der Patientenhistorie und urologischen Indikation liegt es im Ermessen des Arztes, ob die Anamnese synchron oder asynchron erfolgen kann.	93.3%

18	Im Rahmen der telemedizinischen Versorgung können neue Daten gewonnen werden (sowohl synchrone als auch asynchrone Medizin). Diese Daten können einen wichtigen Beitrag zur Real-World Situation von Patienten beitragen und sollten wissenschaftlich genutzt werden.	93.3%
19	Fachgesellschaften (z. B. DGU) sollen aktiv Standards für die telemedizinische Infrastruktur definieren.	100%
20	Fachgesellschaften sollen aktiv an urologiespezifischen Telemedizinguidelines arbeiten.	80.0%
21	Bei der Anwendung von Telemedizin in der Urologie ist der Arbeitsschutz des Arztes, MFA etc genauso wie in der konventionellen Versorgung zu gewährleisten. Dauerhafter Zugang von Patienten zu medizinischen Personal muss eingegrenzt und überdacht werden.	86.7%
